# Preparation and application of caffeic acid imprinted polymer

**DOI:** 10.55730/1300-0527.3572

**Published:** 2023-05-22

**Authors:** Şeyda KARAMAN ERSOY, Esma TÜTEM, Kevser SÖZGEN BAŞKAN, Reşat APAK

**Affiliations:** 1 Division of Analytical Chemistry, Faculty of Pharmacy, Fenerbahçe University, İstanbul, Turkiye; 2 Department of Chemistry, Faculty of Engineering, İstanbul University-Cerrahpaşa, İstanbul, Turkiye; 3 Turkish Academy of Sciences (TÜBA), Ankara, Turkiye

**Keywords:** Caffeic acid imprinted polymer, caffeic acid recovery, chlorogenic acid recovery, green coffee bean, molecularly imprinted solid phase extraction

## Abstract

In the present study, molecularly imprinted polymers were synthesized using caffeic acid (CA) as a template molecule and then used for the extraction of CA and chlorogenic acid (CLA) from complex matrices. Syntheses were carried out in tetrahydrofuran as porogenic solvent using 4-vinyl pyridine, methacrylic acid, acrylamide, and 1-vinyl imidazole as monomers, ethylene glycol dimethacrylate as crosslinker and 2,2′-azobisisobutyronitrile as initiator. In polymerization processes, different ratios of the template:monomer:crosslinker (T:M:CrL) were used to obtain the most suitable polymer. Caffeic acid:4-vinylpiridine:ethylene glycol dimethacrylate’s 1:4:16 mole ratio of MIP was determined as the most convenient polymer for CA recognition. In addition, nonimprinted polymers (NIPs) without templates were prepared. Dynamic and static adsorption tests were applied to determine the absorption features of the NIPs and CA-MIPs. Separation and purification studies of CA and CLA were performed with molecular imprinted solid phase extraction (MISPE) application. All steps of MISPE (loading, washing, elution) were optimized by HPLC analysis.

## 1. Introduction

Hydroxycinnamic acids are derivatives of phenyl-propanoids and are found in plant foods and are phenolic constituents that play a central part in the phenolic metabolism of plants and are biosynthetic derivatives of phenylalanine [1,2]. These components are found in herbal (or plant-based) products such as fruit, vegetables, seeds, coffee, flowers, nuts, wine, tea, and olive oil. CA, p-coumaric acid (*p*-COA), and CLA the quinic acid ester of caffeic acid, are the most abundant and essential hydroxycinnamic acids in fruits such as apples, pears, grapes, and plants. CLA displays carcinogenic, antimutagenic, and antioxidant activities in vitro [3]. CLA which is also known as 5-caffeoylquinic acid is stored as the CA (4).

CA is a phenolic antioxidant found in many herbs and beverages. Seventy percent of the total hydroxycinnamics in fruits is caffeic acid. It is an antioxidant that slows down inflammation and thus has important biological effects such as protection to the free radicals’ harmful influences and endothelial damage [2]. Hence, the isolation and enrichment of caffeic acid is an important research topic, and to achieve this goal, CA imprinted polymers are synthesized by using CA as a template [5–9].

Coffee is a drink which is highly consumed in the world [10]. Green coffee has a large caffeine and polyphenol content. Among the polyphenols, it contains the highest amount of chlorogenic acid, caffeic acid, as well as CA, *p*-COA and ferulic acid (FA). Polyphenol content of coffee changes during the roasting process. A cup of coffee which approximately contains 10 g of coffee has a mean of 15–325 mg CLA [10,11]. Coffee has antioxidant and antineoplastic effects. Green coffee has a light aroma which is similar to green bean [12]. Extracts of green coffee have proven that have antihypertensive effects with studies conducted in recent years. For humans and mice, it has a restrictive impact on fat gathering and the body heaviness and regulates the metabolism of glucose for humans [13]. The caffeine contained in coffee has influences on endocrine system, cardiovascular system, and central nervous system [10].

MIPs are polymers comprising a template that can chemically recognize a particular molecule (or a derivative thereof) [14, 15]. MIPs are among the fields of interest in recent years due to their low cost, superior mechanical power, resistance to pressure and temperature, physical strength, stability in the presence of extreme conditions such as organic solvents, metal ions, acids and bases, and high storage durability [16–21].

The continual requirement for rapid and productive novel ways in environment, biotechnology and medicine has guided investigators to canalize more selective, preferable, and susceptible analytical works [22]. So far, MIPs have been used in a vast majority of analytical applications including but not limited to solid-phase extraction (SPE) [23], liquid chromatography [18, 24], capillary electrochromatography, and capillary electrophoresis [25]. MIP adsorbents are used in the separation of peptides, proteins, amino acids, hormones, DNA and RNA; SPE of drugs; and removal and purification of many substances from foods and solid phase extraction of drugs [6, 23–26].

A few CA imprinted polymers prepared by different researchers and their applications are available in the literature. However, their preparation and application methods are different. When these studies are examined, it is seen that there is still a need for simple, fast, and easy-to-apply studies to be carried out for this purpose. Valero-Navarro et al. [8] synthesized the CA imprinted polymer by precipitation polymerization using 4-vinylpyridine (4-VP) as functional monomer and used it for the extraction of CA from the juice sample. The polymer has been used as an HPLC stationary phase, but in the chromatogram (λ = 274 nm), the tailed peak of CA could be determined in a very wide time interval (approximately 5–17.5 min). Besides, this tailed CA peak highly overlaps with the protocatechuic acid (PCA) and CLA peaks. The same authors pointed out that there were only three studies in which CA was used as a template for MIP synthesis [6, 9, 27]. On the other hand, they stated that their selectivity for separating CA from complex matrices is not very high [8]. In a few recent studies, MIPs were applied to different plant extracts for the isolation of quinic acid and/or its derivatives (CA and CLA) [28,29]. In a study by Miura et al., MIPs for CA have been prepared using 4-VP and methacrylamide (MAM) as functional monomers by precipitation polymerization by using a 0.66 (CA):3 (MAM):3 (4-VP) ratio. This polymer was applied for the extraction of CA and CLA in the leaves of *Eucommia ulmodies*. In this study, the retention and molecular recognition properties of the MIPs have been evaluated using water-acetonitrile, and sodium phosphate buffer-acetonitrile as mobile phases in hydrophilic interaction chromatography (HILIC) and reversed-phase chromatography, respectively. As a result, the MIP showed higher molecular-recognition ability for CA in HILIC mode than in reverse-phase mode [28]. In another study, a quinic acid (QA) imprinted MIP has been prepared with a 1:5 template:monomer (QA:4-VP) ratio and the selectivity of MIP towards QA has been tested versus its analogues found in coffee (CA and CLA) with MISPE. The result has shown a recovery percent of 81.92 ± 3.03, with a significant reduction in the amounts of other components (i.e. CA and CLA) in the extract [29].

The primary aim of this study was to design a new suitable CA imprinted polymer for the separation and purification of CA from a synthetic mixture containing phenolic compounds, and CLA from green coffee bean extract, respectively, and to optimize new MISPE application conditions. For purpose a new template:monomer:crosslinker ratio as 1:4:16 was used for the first time by noncovalent imprinting technique [5, 6, 9, 30, 31]. In polymerization processes, CA was used as a template and monomers, porogens, and ratios of the template:monomer:crosslinker (T:M:CrL) were optimized to obtain the most suitable polymer. Dynamic and static adsorption tests were used for CA-MIPs and NIPs. MISPE trials were performed with synthesized CA-MIPs by filling into SPE cartridges in determined amounts. Thus, MISPE was carried out with a synthetic mixture consisting of antioxidant standards. In this way, all steps of MISPE (loading, washing, elution) were optimized by HPLC so that the highest efficiency of CA recovery could be obtained. Then, a new MISPE method was improved for natural sample extracted with a suitable solvent system. In the CA-MISPE application, CLA was recovered from green coffee bean extract as a natural sample. Therefore, our study enables not only in terms of preconcentration and cleaning of CA and CLA, but also selective extraction of these phenolic compounds in complex or contaminated samples.

## 2. Materials and methods

### 2.1 Chemicals

Acrylamide (AA) and vanillic acid (VA) were obtained from Fluka. Sodium sulfate, methanol (MeOH), gallic acid (GA), 4-hydroxybenzoic acid (4-HBA), 1-vinylimidazole (1-VI), acetic acid (HAc), sinapic acid (SA), CLA, rosmarinic acid (RA), *p*-coumaric acid (*p*-COA), acetonitrile (ACN), ferulic acid (FA), and 3,4-dihydroxybenzoic acid (3,4-diHBA) were purchased from Sigma-Aldrich. CA and catechin hydrate (CAT) were obtained from Sigma. 2,2′-azobisisobutyronitrile (AIBN), quercetin hydrate (QC), ethylene glycol dimethacrylate (EDMA), 4-vinylpyridine (4-VP), methacrylic acid (MAA) were from Aldrich. Fuming (37%) hydrochloric acid, *o*-phosphoric acid, tetrahydrofuran (THF), ethanol (EtOH), dimethyl sulfoxide (DMSO), acetone (AC) were purchased from Merck. All the chemicals (phenolic compounds, monomers, crosslinker, porogens, initiator, etc.) and solvents were in analytical purity forms. The initiator (AIBN) was recrystallized from MeOH at 45 °C and THF was dried with Na and distilled before starting the polymer synthesis.

### 2.2 Preparation of caffeic acid imprinted polymer

For preparation of dried THF, 10 g of benzophenone was transferred into a 1-L flask with 500 mL of THF and the contents were stirred after when 5 g of very finely cut sodium pieces were put into the flask. It was observed that the color of the solvent was between green and blue with the addition of sodium. Then, the mouth of the balloon was closed, and the mixture was left in dark at 25 °C for one night. It was observed that the color of the mixture became purple due to the benzophenone radical anion formed. Anhydrous THF was obtained by distilling the mixture containing this anion, which is an indicator of THF drying [32,33].

HPLC analysis

An HPLC system (Waters Breeze 2, Milford, MA, USA) comprising a PDA detector (Waters 2998), a binary gradient pump (Waters 1525), and a C18 column (4.6 mm × 250 mm × 5 mm) was used for performing the analyses. The brand of column was ACE (Aberden, Scotland, UK). Empower PRO software (Waters Associates, Milford, MA, USA) was utilized for data analysis.

In HPLC analysis, a gradient elution using binary mobile phase consisting of solvent A (MeOH) and solvent B (0.2 percent *o*-H_3_PO_4_ in H_2_O) was used [30,31]. This method was developed in the following order: 20% A for 3 min, 5%–35% A for 3 min, between 16% and 80% A for 5 min, and 22%–100% A for 16 min. Total analysis time is 22 min. In this method, the slope was carried out 2.0 in all steps. The flow rate was applied as 1 mL min^–1^ and the injection volume was 25 μL. The studying wavelengths were 280 nm (for the analyses of gallic acid and catechin derivatives) and 290 and 320 nm (for the analyses of caffeic acid derivatives).

By utilizing these studying conditions, calibration curves were drawn by using peak area *vs* concentration of each antioxidant.

### 2.3. Preparation of caffeic acid imprinted polymers

In this work, CA-MIPs were prepared with the bulk polymerization using CA as a template. Several functional monomers including AA, MAA, 4-VP, and 1-VI, EDMA as the crosslinker, porogens (acetone and THF) and T:M:CrL ratios (1:4:12, 1:4:16, 1:4:20, 1:5:30, 1:6:30, 1:8:40) were used to obtain the best MIP.

**Figure 1:**
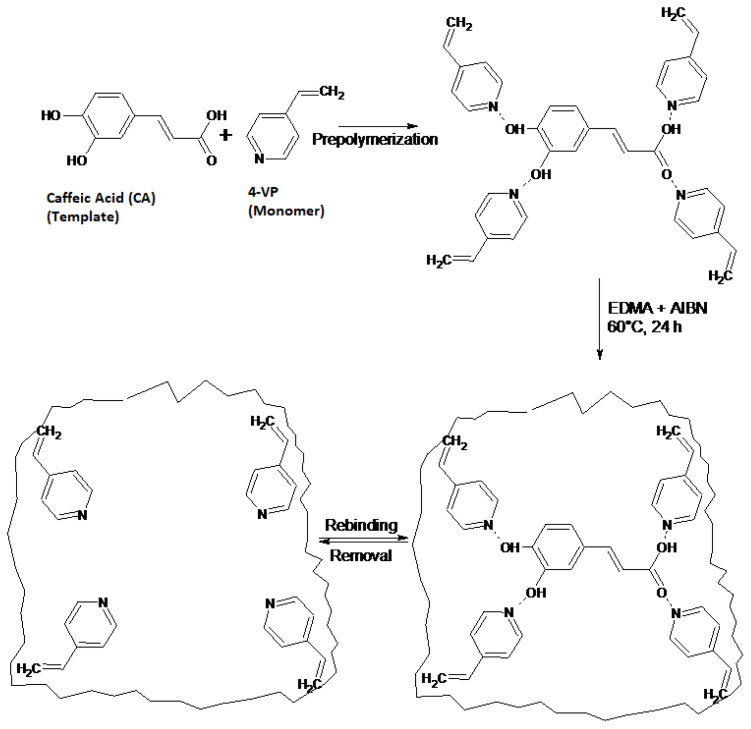
Schematic presentation of the interaction mechanism of MIP.

CA-MIPs and NIPs were synthesized [5, 8, 9, 19, 30, 31] by using 1-VI, 4-VP, AA, MAA and as monomers separately. Figure 1 shows the schematic presentation of the interaction mechanism of MIP. For example, to synthesize the 1:4:16 polymer, in a glass vial 0.4625 mmol of caffeic acid was dissolved in dried THF (9 mL) as porogen, after which in order to prepolymerization 1.85 mmol of monomer was added and mixed for about 10 min. Afterwards, 7.4 mmol of EDMA as crosslinker and 0.93 mmol of AIBN as initiator was put into this mixture. At once nitrogen was passed for 15 min by placing in an ice bath.

After finishing the period, the glass vial was closed and heated in a 60 °C water bath and stayed for 24 h in order to carry out polymerization. Under the same conditions without the use of template molecules; at ratios of nonimprinted polymers with ratios of 0:4:12; 0:4:16; 0:4:20; 0:5:30; 0:6:30; 0:8:40 were synthesized. Afterwards, in order to clear away CA (template), soluble oligomers, and unreacted monomers from the polymer, the product was washed through a Soxhlet extractor by using MeOH-HAc (4:1, v/v) (250 mL × 2) and MeOH (250 mL × 2). Then the polymers were shaken in ACN in a water bath until a steady baseline at UV spectrum of cleaning solvent was acquired. The resulting polymers were milled and sifted to 150–200 μm size of particles in a Retsch Sieve Shaker (Haan, Germany). Then, the drying of the polymers was performed at 50 °C overnight in a vacuum oven (Figure 1).

### 2.4 Adsorption features of CA-MIPs

#### 2.4.1. Time and solvent effects

To determine the time and solvent influences on CA rebinding, batch adsorption tests were used [30]. For this purpose, adsorption solutions were put in a shaker for certain times at room temperature. At the end of these experiments, the obtained supernatant was decanted and filtered? to clarify by using a GF/PET (glass fibre/polyethyleneterephthalate) 1.0/0.45 μm microfilters. The quantities of working compounds of the ending filtrates were established by using their absorbances in their specific wavelengths with the Shimadzu 2600 (Kyoto, Japan) UV-Vis spectrophotometer.

The effect of time was investigated with 4 mL of 40 μM CA in ACN into 30 mg of 1:4:16 CA-MIP in five conical flasks, separately. These mixtures were shaken for 2, 4, 6, 8, 24 h. For defining of solvent effects on rebinding, MeOH, THF, DMF, ACN, and ACN: DMSO (70:30, 90:10, 95:5, 97:3, 98:2, v/v) mixtures were applied. These tests were applied by addition of 4 mL 60 μM CA solutions (arranged separately in above solvents) in 30 mg 1: 4: 16 CA-MIP and NIP.

#### 2.4.2. Adsorption tests

Adsorption experiments consisted of static and dynamic tests [6, 19, 30]. For static tests, 30 mg 1:4:16 CA-MIPs and NIPs were put in conical flasks, separately, then they were blended with 4 mL of CA in certain amounts (20–100 μM) in ACN. At that time these samples were shaken at 250 rpm for 6 h at room temperature.

Dynamic adsorption tests was performed with an Agilent (Waldbronn, Germany) 12-port SPE system and Hamilton (Bonaduz, Switzerland) vacuum pump. For this purpose, 3 mL Hamilton (Bonaduz, Switzerland) empty cartridges were filled with 100 mg of 1:4:16 CA-MIPs by a wet packing method. Then, a polyethylene disc frit was fitted on the top and the bottom of the MIP bed. ACN was used to condition cartridges. Afterwards, 40 μM of CA solution in ACN were loaded into the SPE system at a flow rate of 0.3 mL min^−1^ until releases were detected. The quantities of CA in the effluents were defined were defined by their absorbances at 320 nm. Breakthrough curves were plotted by using concentrations of effluents and volume of sample passed through the CA-MIPs.

#### 2.4.3. Selectivity tests

These studies were applied by using *p*-COA, CLA, SA, FA, RA (hydroxycinnamic acids), VA, 4-HBA, GA, 3,4-diHBA (hydroxybenzoic acids), CAT (a flavanol) for 1:4:16 CA-MIP and 0:4:16 NIP.

Batch tests were carried out in conditions previously stated. Thus 30 mg of 1:4:16 CA-MIP and NIP were added in conical flasks and put in 80 μM (4 mL) of compounds indicated above. The quantities of compounds in the resulting filtrates were defined with their absorbances at max absorption wavelengths in the UV spectra.

**Usage of molecularly imprinted solid phase extraction (MISPE) methods**

Fifty milligrams of 1:4:16 CA-MIP and 1 mL empty SPE cartridge and a polyethylene (PTFE) disc frit that were placed on the bottom and top of the MIP bed were used [6, 30,31]. MIP which was suspended in ACN was filled into this cartridge, afterwards this filled cartridge with MIP was conditioned with ACN. CA and CLA were isolated by using these MISPE methods from the synthetic mixture and green coffee bean extract, respectively. MISPE processes were applied by using loading (flow rate: 0.3 mL min^−1^), washing (flow rate: 1 mL min^−1^) and elution (flow rate: 0.5 mL min^−1^) steps. Before HPLC analyzes of MISPE steps, all eluates were diluted with water at 1:1 (v/v) ratio. Then CA and CLA were analyzed.

### 2.5. MISPE application for the synthetic mixture

For the isolation of CA from synthetic mixture with CA-MISPE application [6, 30]; after cartridge conditioning with ACN, the mixture was loaded in the cartridge with the total volume of 1.5 mL (0.5 mL parts of mixture was loaded in all loading steps). Then washing processes were performed comprising 2 stages with ACN (2.5 mL+0.5 mL) targeting to eliminate compounds that were held by the polymer nonspecifically. Finally, the elution step was applied with MeOH:HAc (4:1,v/v) in 4 stages (each stages comprising 0.5 mL solvent).

### 2.6. MISPE application for the green coffee bean

For the isolation of CLA from green coffee bean extract with CA-MISPE application; firstly, the green coffee bean extract was obtained with aqueous 70% (v/v) methanol as the extraction solvent [30, 31]. One and six-tenths grams of ground green coffee beans were put in stoppered flasks and 70% (v/v) methanol was added. Then the mixture was put in an ultrasonic bath. Extraction was carried out in three stages and took for about 120 min with aqueous 70% (v/v) MeOH as extraction solvent. In order of, 10 mL solvent for 60 min, 10 mL solvent for 45 min and 5 mL solvent for 15 min. Finally, every solvent fraction was collected and fulfilled to 25 mL. Then, the obtained extract of 20 mL was evaporated by using a Büchi R210/215 (Flawil, Switzerland) rotary evaporator at 40 °C under vacuum. After this process, the residue was dissolved in 7 mL of ACN:DMSO (98:2, v/v). One milliliter of that solution was diluted to 4 mL with ACN and dried with anhydrous Na_2_SO_4_.

For MISPE application, 3.5 of the dried green coffee bean extract was loaded into 100 mg of 1:4:16 CA-MIPs conditioned with 10 mL ACN in 3 mL SPE cartridge. Washing steps consisted of 2 steps (12+8 mL ACN) in 1 mL min^−1^ flow rate. Elution was applied by using MeOH:HAc (4:1,v/v) in 0.5 mL min^−1^ flow rate for two successive times consisting of 3 mL and 2 mL, respectively. Afterwards, the residue was dissolved with 7 mL of ACN:DMSO (98:2, v/v). One milliliter of this extract was diluted with ACN to 4 mL and dried with anhydrous Na_2_SO_4_. Three and five-tenths milliliters of the dried green coffee bean extract was loaded into 100 mg of 1:4:16 CA-MIPs conditioned with 10 mL ACN by using 3 mL SPE cartridge. Washing steps comprised 2 steps (12 + 8 mL ACN) in 1 mL min^−1^ flow rate. The elution process was performed with MeOH:HAc (4:1,v/v) in 0.5 mL min^−1^ flow rate for 2 times as 3 mL and 2 mL.

## 3. Results

### 3.1. Arranging of MIP synthesis

These studies were evaluated with imprinting factors (Eq. 1) by batch tests with utilizing 60 mM CA and 30 mg MIP and NIP. To determine the most suitable monomer for polymer synthesis, MIPs and NIPs were synthesized in 1:4:16 ratio of template:monomer:crosslinker using four several monomers comprising MAA, AA, 1-VI, and 4-VP. The imprinting factors (IFs) of the polymers were estimated with adsorption experiments using 60 μM CA with these polymers (Eq. 1). The results are shown in Table 1. CA adsorption did not occur in polymers synthesized by using AA as monomer, and CA adsorption was higher in NIPs than MIP in polymers synthesized using 1-VI and MAA. This is an indication that CA does not form in MIP and that adsorption occurs nonspecifically. With polymers synthesized by using 4-VP as monomer, more CA was adsorbed in MIP and NIP compared to other monomers, and since the adsorption on MIP was higher than NIP, the formation of CA in MIP was confirmed in this way and imprinting was achieved.


(1) $$\text{Imprinting factor }(\text{IF})={\text{Q}}_{\text{MIP}}/{\text{Q}}_{\text{NIP}}$$

Q_MIP_: adsorption amount of MIP (μg g^−1^)
Q_NIP_: adsorption amount of NIP (μg g^−1^)

Syntheses were made using methanol, ACN and THF as porogenic solvents for CA-MIPs, but it was observed that adsorption occurred in the polymer synthesized in THF. The imprinting factor (IF) for the polymer prepared with this solvent was calculated as 2.02 since the amount of CA adsorbed by CA-MIP is 930 and NIP has 460 mg g^−1^. Also, the availability of polar solvents like water disturbs template and monomer interaction outcoming in polymers with a weak grade of identification. Because of this reason, THF is dried with Na (32, 33).

In the syntheses of caffeic acid imprinted polymers, using different T:M:CrL mole ratios (1:4:12, 1:4:16, 1:4:20, 1:5:30, 1:6:30, 1:8:40) CA-imprinted and nonimprinted polymers with 4-VP monomer and THF solvent. In Table 1, polymer mole ratios and CA adsorptions are compared. The highest imprinting factor (2.02) was obtained with the 1:4:16 polymer. For this reason, it was concluded that the most suitable template molecule:monomer:crosslinker molar ratio is 1:4:16. Table 1

### 3.2. Adsorption characteristics of CA-MIPs

#### 3.2.1. Time and solvent impacts on rebinding

In order to examine time effect, for batch rebinding tests, 6 h was approved of shaking period as adequate. To the adsorption quantity of CA vs. hour did not indicate a clear alteration later 4 h. At the 6th hour, it achieved a plateau.

In order to examine solvent influence, usage of ACN:DMSO (70:30, 90:10, 95:5, 97:3, 98:2, v/v), ACN: H_2_O (50:50, v/v), MeOH, ACN, THF, DMF mixtures was performed on batch adsorption tests.

It was observed that CA solutions at 40 mM concentration were not adsorbed in MeOH, THF, DMF and ACN:DMSO (70:30, 90:10, 95:5, v/v) solvent mediums. As can be seen in Table 2, ACN and ACN: DMSO (97:3, 98:2, v/v) solvents mediums were suitable for batch adsorption tests. Although imprinting factor was the highest in ACN:DMSO (98:2, v/v) solvent medium, in CA-MISPE experiments DMSO ratio was kept low by using ACN in dilution solvent. Table 2

#### 3.2.2. Adsorption tests

**Figure 2:**
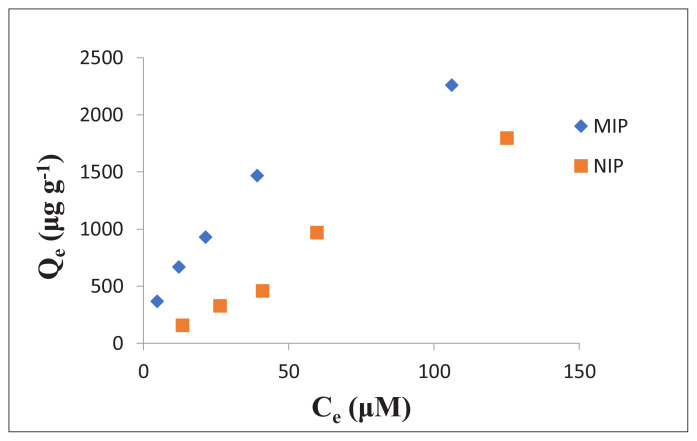
Adsorption isotherms belonging to 1:4:16 CA-MIP and NIP (C_e_: equilibrium CA concentration, mM; Q_e_: amount of adsorbed CA of polymer, mg g^−1^).

To determine impact of CA concentration on adsorption capacity, 30 mg 1:4:16 CA-MIP and various concentrations of CA (0.02–10.00 mM) in ACN were used in static adsorption experiments. Table 3 indicates the adsorption amounts of CA and imprinting factors, Figure 2 demonstrates adsorption isotherms for imprinted and nonimprinted polymers [9]. As can be seen from Table 3, it was determined that although imprinting factor was elevated at low CA concentration, considerably low at high CA concentration. In addition, the amount of CA adsorbed by NIP at concentrations below 0.40 mM is lower than MIP. But the amount of CA adsorbed by NIP at a 0.40 mM CA approaches MIP adsorptivity and even exceeds MIP adsorption at higher concentrations. It is thought that the reason for this is the low solubility of CA in acetonitrile, and therefore, at high concentrations, CA collapses over time. Therefore, it was concluded that at high concentrations, the measured absorbances could not be related to the polymer’s binding to CA. The imprinting factor also decreases rapidly above 0.06 mM CA concentration. For these reasons, concentrations above 0.20 mM were not used in adsorption studies (Table 3).

**Figure 3:**
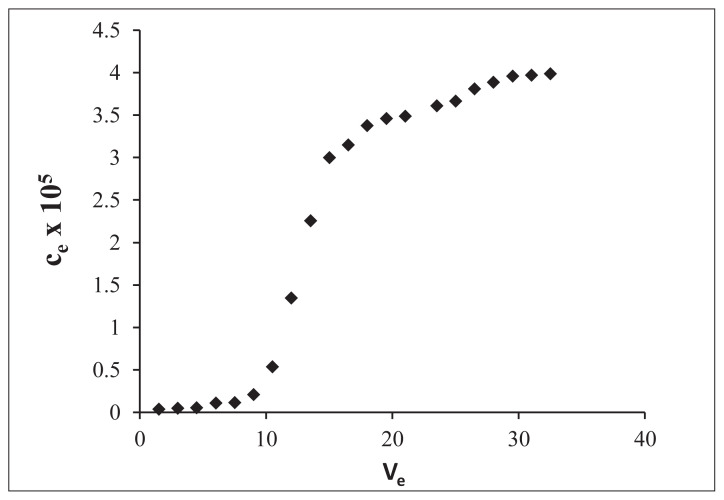
Breakthrough curve of 1:4:16 CA-MIP in ACN (C_e_: equilibrium CA concentration, mM; V_e_: effluent volume, mL).

In dynamic adsorption tests (column experiments), breakthrough curve was plotted with CA concentration (C_e_) vs. the volume (V_e_) of effluent in ACN solvent (Figure 3). The dynamic adsorption capacity of 1:4:16 CA-MIP was determined with calculation by the integration the area above breakthrough curve was 5.7 × 10^−3^mmol (1.03 mg g^−1^).

#### 3.2.3. Adsorption isotherms of CA-MIP and NIP

Adsorption features of 1:4:16 CA-MIP and NIP were estimated by using Freundlich and Langmuir isotherms.

The linear form of Freundlich isotherm is explained as Eq. 2:


(2) $${\text{lnQ}}_{\text{e}}={\text{lnK}}_{\text{f}}+1/\text{n }{\text{lnC}}_{\text{e}}$$

Q_e_: adsorption amount of CA on MIP and NIP (mg g^−1^)
C_e_: equilibrium concentration (concentration remaining in solution at equilibrium, mM).

**Figure 4:**
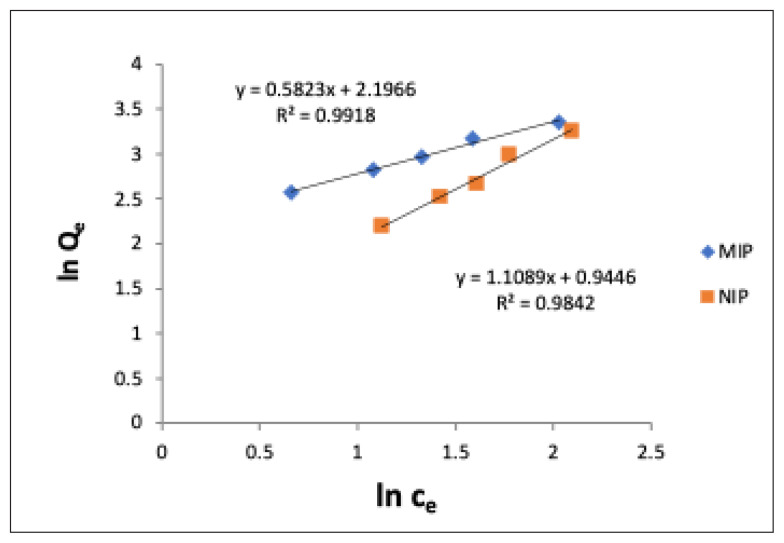
Linearized Freundlich isotherms of 1:4:16 CA-MIP and NIP (C_e_: CA concentration at equilibrium, μM; Q_e_: adsorption amount of polymer, μg g^−1^).

It was concluded that the adsorption complies with the Freundlich isotherm due to the high coefficient of agreement for CA in CA-MIP and the n number being >1 (Table 4; Figure 4).

**Figure 5:**
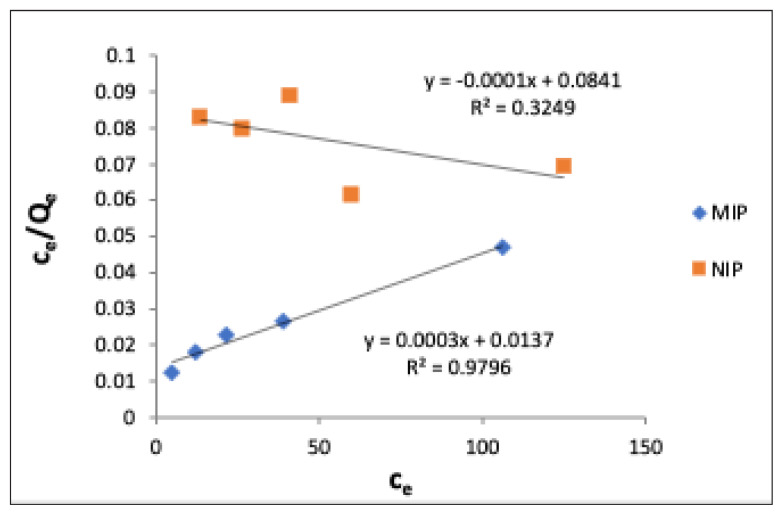
Linearized Langmuir isotherms of 1:4:16 CA-MIP and NIP (C_e_: equilibrium CA concentration μM; Q_e_: amount of adsorbed CA of polymer, μg g^−1^).

Linear Langmuir adsorption isotherm was drawn according to Eq. 3 between c_e_ and c_e_/q_e_ values for CA in the same CA-MIP and NIP (Figure 5) and calculation of q_max_ was performed by using the slope of the obtained line, and b values were calculated from the shift value. Table 5 shows the magnitudes of the Langmuir isotherm of MIP and NIP.


(3) $${\text{Q}}_{\text{e}}={\text{Q}}_{\text{max}}\;\text{b }{\text{C}}_{\text{e}}/(1+\text{b }{\text{C}}_{\text{e}})$$

(4) $${\text{C}}_{\text{e}}/{\text{Q}}_{\text{e}}=1/({\text{Q}}_{\text{max}}\;\text{b})+{\text{C}}_{\text{e}}/{\text{Q}}_{\text{max}}$$

It has been concluded that the Freundlich isotherm is more suitable for this MIP, since the coefficients fit obtained for CA adsorption in CA-MIP are lower than those obtained with the Freundlich isotherm and the q_max_ value is much larger than the experimentally found one.

**Selectivity tests**

**Figure 6:**
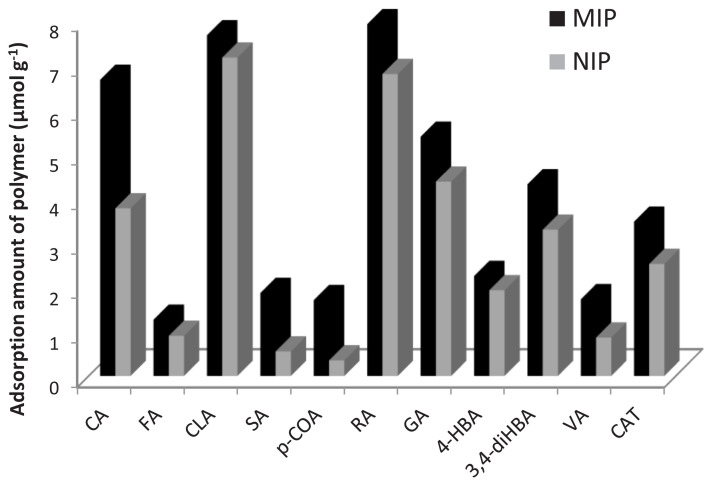
Adsorption selectivity of phenolic compounds for CA-MIP and NIP.

Selectivity tests for 1:4:16 CA-MIP and NIP were carried out by utilizing 80mM of sinapic acid (SA), p-coumaric acid (p-COA), ferulic acid (FA), chlorogenic acid (CLA), rosmarinic acid (RA), from the hydroxycinnamic acid class such as caffeic acid (CA); 4-hydroxybenzoic acid (4-HBA), gallic acid (GA), prothocatechuic acid (PA), 3,4-dihyroxybenzoic acid (3,4-diHBA), vanillic acid (VA) from the hydroxybenzoic acid class, catechin (CAT) form the flavonoids class of solutions, respectively. The reason for using these antioxidant standards in selectivity studies is to determine specific and nonspecific adsorptions by testing the selectivity of the polymer we obtained for antioxidants from the phenolic acid and flavonoid class, which are mostly found in plant extracts. Figure 6 indicates the adsorption quantities of MIP and NIP. Thus, it has been often observed that analytes can be retained by MIPs and NIPs through nonspecific interactions assisted mainly by solvophobic effects [34]. Due to nonspecific ionic interactions, the adsorption amounts obtained by MIP and NIP are close to each other because these interactions are not specific to MIP. Since such adsorptions are not specific, they can be easily removed from the polymer with suitable solvent systems during the washing steps in MISPE studies. Figure 6

The increasing adsorption amount of phenolics (μmol g^−1^) for MIP was as follows: FA (1.26) < *p*-COA (1.70) < VA (1.72) < SA (1.86) < 4-HBA (2.24) < CAT (3.46) < 3,4-diHBA (4.30) < GA (5.37) < CA (6.65) < CLA (7.65) < RA (7.90).

The increasing adsorption amount of phenolics (μmol g^−1^) for NIP was as follows:

*p*-COA (0.35) < SA (0.55) < VA (0.86) < FA (0.90) < 4-HBA (1.92) < CAT (2.51) < 3,4-diHBA (3.28) < CA (3.76) < GA (4.36) < RA (6.78) < CLA (7.11).

### 3.3. CA-MISPE applications

#### 3.3.1. Synthetic mixture

**Figure 7:**
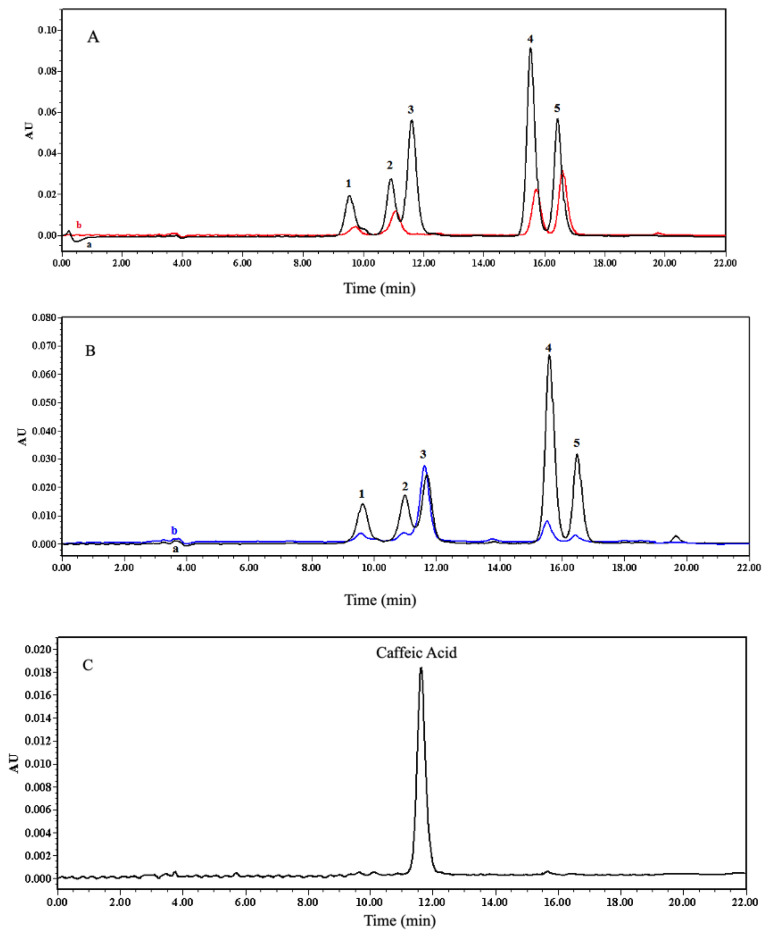
Chromatograms of A) (a) before and (b) after sample loading into the MISPE cartridge, B) washing steps of MISPE [a: washing 1: 2.5 mL ACN, b: washing 2: 0.5 mL ACN], C) elution step of MISPE [0.5 mL MeOH:HAc (4:1, v/v)] (λ: 280 nm) (1: VA, 2: CAT, 3: CA, 4: *p*-COA, 5: FA).

One and five-tenths milliliters mixture in ACN comprising 3 × 10^−4^ M VA, CA, *p*-COA, FA, CAT was loaded into the 1-mL SPE cartridge packed with 50 mg 1:4:16 CA-MIP at a flow rate of 0.3 mL min^−1^. Figure 7 shows the chromatograms of all MISPE step solutions.

Figure 7A shows other phenolic compounds, mostly CA, were also retained in the cartridge. *p*-COA and FA have hydroxycinnamic acid structures like CA, whereas CAT is among the flavanol class of flavonoids. But CAT has a 3,4-dihydroxyphenyl structure similar to that of CA. Moreover, VA is a small molecule derivative of hydroxybenzoic acid. Therefore, such structural properties of these compounds cause some nonspecific adsorptions by the polymer. However, since these retentions are expected to be weaker than CA, which is the template molecule, it is aimed to remove these compounds from MIP by washing with suitable solvent systems. Figure 7B indicates the chromatograms of the washing solutions. In this chromatogram, some CA also moves away from the MIP with the washing steps. After the washing step, CA retained in the polymer was recovered with MeOH:HAc (4:1, v/v). Figure 7C shows the elution chromatogram. Other compounds were removed from the cartridge in the washing step and CA recovery was achieved in the elution step with a slightly lower yield (49%) (Figure 7C).

#### 3.3.2. MISPE application of green coffee bean extract with CA-MIP

**Figure 8:**
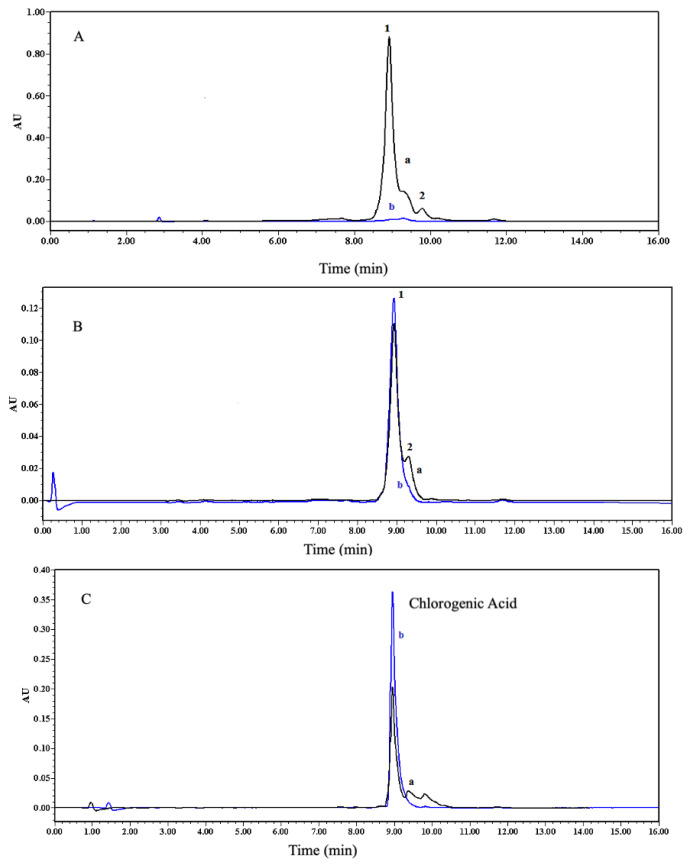
Chromatograms of A) (a) before and (b) after sample loading into cartridge, B) washing steps of MISPE [a: washing 1 (12 mL ACN); b: washing 2 (8 mL ACN)], C) elution steps of MISPE [a: elution 1(3 mL MeOH:HAc (4:1, v/v)); b: elution 2 [2 mL MeOH:HAc (4:1, v/v)]. (λ = 320 nm) (1: chlorogenic acid; 2: caffeine).

After evaporation, 1 mL of extract dissolved in ACN:DMSO (98:2, v/v) was taken and diluted to 4 mL with ACN. Three and five-tenths milliliters of this extract, which was dried with anhydrous Na_2_SO_4_, was taken and loaded into a 3-mL SPE cartridge containing 100 mg 1:4:16 CA-MIP conditioned with 10 mL ACN at a rate of 0.3 mL min^−1^. Figure 8A compares the chromatograms of the extract before and after loading. The detection wavelength was 320 nm, which is close to the maximum adsorption of CLA.

As can be seen, other components were retained in the colon besides chlorogenic acid. Since components other than chlorogenic acid are not specifically retained in CA-MIP, it is aimed to remove them from the cartridge by washing with suitable solvent systems. Figure 8B shows the chromatograms of the washing solutions taken from the column. For green coffee bean extract, as can also be seen in these chromatograms, CLA showed the strongest affinity to the CA-MIP, but caffeine was adsorbed onto the CA-MIP in small amounts. This result was thought, because of the abilities of phenolic compounds to create hydrogen bonds by their -OH groups without entering the cavities of the MIP and nonspecific ionic interactions with the heterogeneous binding sites of the MIP. Using those benefits, caffeine was removed by the washing processes. Therefore, the elution process was applied with 3 mL and 2 mL of MeOH: HAc (4:1, v/v) as two steps at a flow rate of 0.5 mL min^−1^ (Table 6).

## 4. Discussion

The 4-VP monomer was preferred for the preparation of CA-MIPs because the IF value for CA-MIP prepared with this monomer was determined higher than those prepared with other monomers (MAA, AA, 1-VI). This result can be explained by the favorable interactions between basic monomer 4-VP (pKa 5.62) and the acidic template CA (pKa 4.62) [35]. Indeed, many other CA-MIP studies have reported that this monomer is more suitable [5, 8, 9]. The porogenic solvent has a very important role in noncovalent interactions of polymer structure. Because the porogen provides the generation of specific cavities for the template. On the other hand, the polarity of the porogen also affects imprinting. Therefore, moderately polar solvents such as THF will positively affect the imprinting factor of CA-MIPs. Indeed, it was determined THF offers a preferred imprinting factor for CA-MIP. This situation may be due to a competition between the interaction of CA and 4-VP and their interaction with porogen [36] Since methanol has a high polarity and –OH group that can hydrogen bond with caffeic acid, the tendency of CA to hydrogen bond with the monomer during polymerization decreases and the pore formation of CA cannot occur during polymerization. When ACN is used, it is necessary to heat it to ensure dissolution owing to very little CA dissolution in ACN, and since the amount of CA used during polymerization is high, some of the CA remains insoluble, which prevents the formation of CA pore during polymerization. Therefore, it was decided that THF is the most suitable porogenic solvent in synthesis of CA-MIPs. It was stated that in a CA-MIP study the THF is the most suitable porogenic solvent [9]. The highest imprinting factor was obtained at a ratio of 1:4:16 (T:M:CrL). In the selectivity experiments with CA-MIP and NIP, the adsorbed amounts of many phenolic substances were compared besides CA and its quinic acid ester CLA. According to Figure 6, the order of the investigated phenolic substances according to the amount adsorbed on CA-MIP is as follows: FA < *p*-COA< VA< SA< 4-HBA< CAT< 3,4-diHBA < GA < CA < CLA < RA. The order of the same compounds according to the amount adsorbed on the NIP is as follows: *p*-COA < SA < VA < FA < 4-HBA < CAT < 3,4-diHBA < CA < GA < RA < CLA. Since in the case of the NIPs there are no specific sites present, the interactions are mainly of ionic nature, and thus, nonspecific. According to Michailof et al. [9], the interaction differences between MIP and phenolic compounds can be explained by their molecular structures—namely, the sizes and shapes of the molecules, and the presence of double bonds, hydroxyl, carboxyl, or methoxy groups in the molecule. Valero-Navaro et al. [8] have reported that MIP imprinted with CA showed very high selectivity for CLA. They have stated that since CLA is an ester formed between CA and QA, similar shape-selective interactions have been expected for both CA and CLA although stronger retention of CLA than CA can be explained by its high polarity and multifunctionality. On the other hand, Li et al. [6] have shown the opposite result and reported that CLA was retained weaker in their MIPs than CA and other structurally similar compounds (VA, and GA). In this study, it was observed that CLA was first separated from the polymer column when acetonitrile containing 2% acetic acid was used as the eluent, due to its weak retention of it on the monolithic stationary phase of the polymer. In our study, considering the adsorption of FA, which is in the structure of hydroxycinnamic acid, it is seen that although it is similar in structure to CA, the presence of 3-methoxy structure in the phenyl group creates a steric hindrance and reduces the hydrogen bonding efficiency; therefore, its adsorption in MIP and NIP is not very high. Since CLA is the quinic acid ester of CA, it is expected to create a steric barrier to enter the MIP mold, but because it contains too many hydroxyl groups, it is highly adsorbed by both MIP and NIP. Since RA is the phenyl ester of CA and contains many hydroxyl groups, it is highly adsorbed in CA-MIP and NIP. Since the adsorption distinction between MIP and NIP of highly adsorbed CLA and RA is lower than that of CA, the adsorption of these compounds is not as specific as CA. Adsorption of SA is determined less on MIP and NIP due to the steric hindrance of the 3,5-dimethoxy group on the phenyl ring. Unlike CA, *p*-COA also contains only 4-hydroxy structure in the phenyl ring; therefore, its adsorption is low in MIP and NIP. Since GA and 3,4-diHBA, which are in the structure of hydroxybenzoic acid, have a smaller molecular structure than CA and contain 3,4-dihydroxy structure like CA, their retention rate in the polymer is high. The adsorption of VA and 4-HBA by the polymer is low, as they have a single mold attachment point like the CA structure. CAT, which has a flavonoid structure, seems to be adsorbed to some extent by the polymer, since CA and 3,4-dihydroxyphenyl structure are common.

## 5. Conclusion

In this work, synthesis of CA-MIPs was aimed to isolate, purify and preconcentrate CA and CLA from synthetic mixture and natural extracts. These compounds are among the hydroxycinnamic acid class of phenolic acids, and thus, they have important biological effects such as slowing down inflammation and protection against the harmful effects of free radicals.

Therefore, CA-MIPs were obtained by noncovalent bulk polymerization method and adsorption features (recognition and selectivity) of the polymers were determined by binding experiments with CA and several phenolic compounds.

MISPE applications were performed to preconcentrate and purify CA from synthetic mixtures and CLA from coffee bean extract for the first time.

At the end of these experiments, recovery yield of CA was 42% and CLA 49%. This investigation demonstrates that obtained CA-MIP can ensure sufficient extraction CA from complex matrices. In addition, one of the most important advantages of the synthesized new mole ratio of MIP (1:4:16) and the applied new MISPE method is to facilitate and speed up the following chromatographic analysis as sample preparation (clean up) material. Thus, our study enables not only in terms of preconcentration and cleaning of CA and CLA, but also selective extraction of these phenolic compounds in complex or contaminated samples by using new ratio 1:4:16 of MIP in the literature.

## Figures and Tables

**Table 1:** Estimation of CA-MIPs and NIPs (in parenthesis) obtained with several molar ratios, monomers and porogens.

Mole ratio	Monomer	Porogen	Quantity of adsorbed CA^a^ (μg g^−1^ )	Imprinting factor (IF)
1:4:12	4-VP	THF	860 ± 14 (650 ± 25)	1.32
1:4:16	4-VP	THF	930 ± 11 (460 ± 13)	2.02
1:4:20	4-VP	THF	600 ± 17 (570 ± 22)	1.05
1:5:30	4-VP	THF	610 ± 15 (340 ± 18)	1.83
1:6:30	4-VP	THF	510 ± 16 (340 ± 24)	1.50
1:8:40	4-VP	THF	600 ± 14 (430 ± 33)	1.40
1:4:16	AA	THF	0	0
1:4:16	MAA	THF	45 ± 3 (48 ± 9)	0.94
1:4:16	1-VI	THF	655 ± 16 (785 ± 19)	0.87

a Values indicate mean ± SD, n = 3.

**Table 2:** CA adsorption in different solvent medium for 1:4:16 MIP and NIP.

Solvent	Adsorption amount of CA^a^ (μg g^−1^)	Imprinting factor (IF)
ACN:DMSO (98:2, v/v)	340 ± 13 (90 ± 8)	3.78
ACN:DMSO (97:3, v/v)	450 ± 24 (320 ± 29)	1.41
ACN	670 ± 21 (330 ± 18)	2.03

a Values indicate mean ± SD, n = 3.

**Table 3:** Adsorption amount of CA on 1:4:16 CA-MIP and NIP and imprinting factors (IFs) with several concentration of CA.

CA concentration (mM)	Adsorption amount of CA^a^ (mg g^−1^)	Imprinting factor (IF)

0.02	0.370 ± 0.025 (0.160 ± 0.036)^b^	2.31
0.04	0.670 ± 0.021 (0.330 ±0.018)	2.03
0.06	0.930 ± 0.011 (0.460 ± 0.013)	2.02
0.10	1.470 ± 0.014 (0.970 ± 0.018)	1.52
0.20	2.260 ± 0.023 (1.800 ± 0.029)	1.26
0.40	3.670 ± 0.026 (3.170 ± 0.029)	1.16
0.60	4.400 ± 0.038 (5.400 ± 0.045)	0.81
1.00	6.570 ± 0.053 (6.850 ± 0.061)	0.96
10.00	56.670 ± 0.049 (63.330 ± 0.058)	0.89

a Values shows mean ± SD, n = 3.

b Values in parentheses are the amounts of CA adsorbed by NIP.

**Table 4:** Freundlich adsorption isotherm values of MIP and NIP for CA.

Polymer	Freundlich constants		
	K_f_	n	R^2^
**MIP**	157	1.72	0.9918
**NIP**	9	0.90	0.9842

**Table 5:** Langmuir adsorption isotherm values of CA-MIP and NIP for CA.

Polymer	Langmuir constants		
	q_max_	b	R^2^
**MIP**	3333	21.91 × 10^−3^	0.9796
**NIP**	10000	1.19 × 10^−3^	0.3249

**Table 6:** CA-MISPE outcomes for synthetic mixture and green coffee bean extract.

Sample	Amount of CA (mmol)	Amount of CLA (mmol)

Synthetic mixture		
Loaded	2.86 × 10^−4^	-
Adsorbed	2.84 × 10^−4^	-
Recovered	1.40 × 10^−4^ (49%)^a^	-
Green coffee bean extract		3.11 × 10^−3^
Loaded	-	3.01 × 10^−3^
Adsorbed	-	5.22 × 10^−4^ (17%)^a^
Recovered	-	7.48 × 10^−4^ (25%)^a^

a Recovery %.
